# KickStat: A Coin-Sized Potentiostat for High-Resolution Electrochemical Analysis

**DOI:** 10.3390/s20082407

**Published:** 2020-04-23

**Authors:** Orlando S. Hoilett, Jenna F. Walker, Bethany M. Balash, Nicholas J. Jaras, Sriram Boppana, Jacqueline C. Linnes

**Affiliations:** 1 Weldon School of Biomedical Engineering, Purdue University, West Lafayette, IN 47907, USA; ohoilett@purdue.edu (O.S.H.); walke327@purdue.edu (J.F.W.); bmdoehrm@purdue.edu (B.M.B.); boppana113@gmail.com (S.B.); 2 School of Electrical and Computer Engineering, Purdue University, West Lafayette, IN 47907, USA; njaras24@gmail.com

**Keywords:** wearable, point-of-care, open-source, voltammetry, biosensors, electrochemistry, amperometric, low-cost, miniaturized

## Abstract

The demand for wearable and point-of-care devices has led to an increase in electrochemical sensor development to measure an ever-increasing array of biological molecules. In order to move from the benchtop to truly portable devices, the development of new biosensors requires miniaturized instrumentation capable of making highly sensitive amperometric measurements. To meet this demand, we have developed KickStat, a miniaturized potentiostat that combines the small size of the integrated Texas Instruments LMP91000 potentiostat chip (Texas Instruments, Dallas, TX, USA) with the processing power of the ARM Cortex-M0+ SAMD21 microcontroller (Microchip Technology, Chandler, AZ, USA) on a custom-designed 21.6 mm by 20.3 mm circuit board. By incorporating onboard signal processing via the SAMD21, we achieve 1 mV voltage increment resolution and an instrumental limit of detection of 4.5 nA in a coin-sized form factor. This elegant engineering solution allows for high-resolution electrochemical analysis without requiring extensive circuitry. We measured the faradaic current of an anti-cocaine aptamer using cyclic voltammetry and square wave voltammetry and demonstrated that KickStat’s response was within 0.6% of a high-end benchtop potentiostat. To further support others in electrochemical biosensors development, we have made KickStat’s design and firmware available in an online GitHub repository.

## 1. Introduction

Current electrochemical biosensors are capable of detecting a wide range of analytes such as lactate [1,2], sodium [2,3,4], cocaine [5,6], alcohol [7] and, most famously, glucose [8] for managing diabetes. Such electrochemical measurements are performed using potentiostats, instruments containing the necessary features for accurate measurements of the electrochemical cell [9,10,11]. Recently, potentiostats for simple amperometric sensing of glucose, lactate, and sodium, have been miniaturized into sweat-analyzing wearables [2]. In contrast, emerging surface-based electrochemical sensors, such as nucleic acid aptamer-based biosensors, have shown promise in extending the available molecules for detection [5,12]. However, surface electrochemistry with bound aptamers and antibodies requires more sophisticated detection modalities than solution electrochemistry used for sugars and salts [8,13]. Such aptamer-based biosensors require pulse voltammetry techniques like square wave voltammetry (SWV) [13] to obtain the necessary sensitivity to accurately quantify the signal produced by the aptamer upon binding to the analyte.

Due to the growing popularity of electrochemical biosensors, numerous in-house potentiostats have been presented in the literature [9,14,15,16,17,18,19,20,21,22,23,24,25,26,27]. Such designs aim to decrease the cost and increase the accessibility of potentiostats with the goal of democratizing scientific research or to miniaturize electrochemistry hardware for use in at-home or portable diagnostic devices. However, such designs require extensive hardware expertise in order to replicate and utilize too many discrete components for wearable sensors and other applications where extreme portability is a priority. To address the growing needs for miniaturization, improvements in semiconductor fabrication have allowed for the development of potentiostats down to single integrated circuits (ICs) measuring just a few square millimeters [14,28,29,30]. One such IC, the LMP91000 developed by Texas Instruments, contains the core features of a benchtop potentiostat including a 14-point setting voltage reference, ohmic drop compensation, and a transimpedance amplifier in a 4 mm × 4 mm package [14,29]. The LMP91000’s integrated features allow for basic electrochemical techniques such as cyclic voltammetry (CV) and chronoamperometry (CA) used in lactate and glucose sensors, but lack sufficient voltage reference generators to perform more complex voltammetric techniques like SWV and normal pulsed voltammetry (NPV). Some designs have leveraged the LMP91000 [7,14,22,23,24,25,26,31,32], but have limited voltage reference generators and limited, if any, on-chip signal-processing capabilities.

Here, we adapt the traditional LMP91000 to include onboard signal processing and advanced electrochemical capabilities, improving upon the work done by others utilizing this same IC. With minimal external components we can improve the LMP91000’s capabilities to achieve a voltage reference generator with 1 mV resolution, and current limit of detection down to 4.5 nA, thus enabling complex electrochemical techniques such as SWV and NPV. We demonstrate these capabilities by first analyzing a 5 mM solution of potassium ferricyanide, an ideal Nernstian redox couple, in order to characterize our device performance using popular electrochemical techniques such as CV, SWV, NPV, CA. We then measure the response of an electroactive DNA biosensor for cocaine. The cocaine biosensor produces nanoamps of current and requires a voltage reference generator with a resolution of 1 mV to accurately quantify the faradaic current [13,27]. Electrochemical biosensors are increasing in popularity, and their response current is often very small. The LMP91000 is not able to make such measurements with its stock capabilities, making our improvements necessary to maximize the IC’s utility to the growing biosensors space. Our design supports the democratization of research by providing a minimalistic, yet versatile solution for applications where miniaturization is a priority, such as wearable sensors and handheld diagnostic devices. Finally, our hardware designs and source code are publicly available in an online GitHub repository [33], making our work accessible and reproducible to the larger scientific audience.

## 2. Materials and Methods

### 2.1. Reagents and Chemicals

All solutions were dissolved in ultrapure water obtained from a Millipore Milli-Q (MilliporeSigma, St. Louis, MO, USA) system unless specified otherwise. Phosphate-buffered saline (PBS) was purchased from MilliporeSigma (Product ID: P-5368, St. Louis, MO, USA) and prepared at a concentration of 0.01 M PBS at pH 7.4 and room temperature. Sulfuric acid was purchased from MilliporeSigma (St. Louis, MO, USA) and diluted to 0.5 M in ultrapure water. Potassium ferricyanide was purchased from MilliporeSigma (St. Louis, MO, USA) and dissolved in PBS buffer solution to create a 5 mM solution. Alumina polishes for preparing the electrode were purchased from CH Instruments Inc. (Austin, TX, USA).

The anti-cocaine aptamer sequence (5’ HSC11-AGACAAGGAAAATCCTTCAATGAAGTGGG- TCG-MB 3’) was obtained from the literature [5] and purchased from LGC Biosearch Technologies (Petaluma, CA, USA). The sequence was modified with an 11-carbon thiol group (HSC11-) on the 5’ end of the DNA sequence and an electroactive methylene blue (-MB) compound on the 3’ end. The aptamer was provided as a dried lyophilized pellet and was dissolved in ultrapure water to create a 200 μM solution, which was stored in the dark at 4 °C until use. Cocaine hydrochloride was purchased from MilliporeSigma (St. Louis, MO, USA) in powdered form following US Drug Enforcement Agency approved protocols for handling controlled substances. Up to 3 mg was dispensed using an analytical balance (UMX5 Comparator, Mettler Toledo, Columbus, OH, USA) and dissolved in appropriate volume of PBS to create a 0.5 mM solution. Cocaine hydrochloride solution was made fresh for each day’s experiments and disposed of following protocols for controlled substances.

### 2.2. KickStat LL

**Figure 1:**
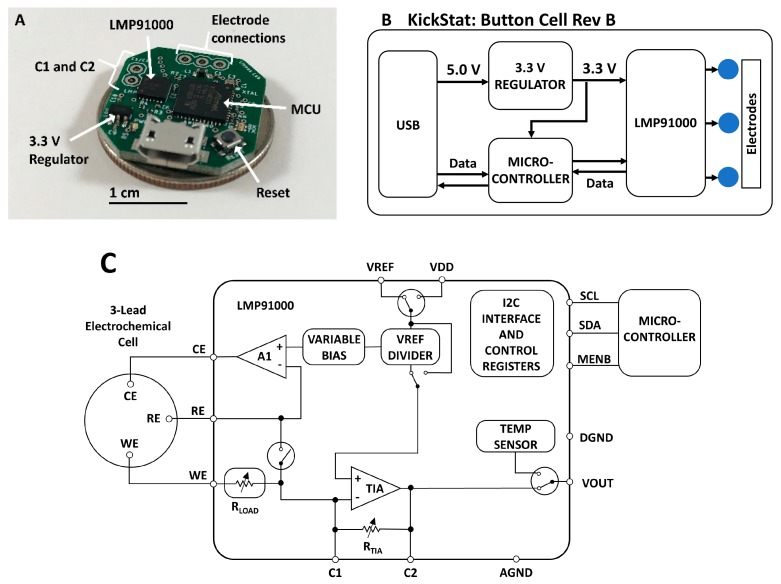
(**A**) Photograph of the assembled KickStat: Button Cell Rev B. The device features the LMP91000 along with a SAMD21 microcontroller running an Arduino bootloader, (**B**) functional block diagram of KickStat: Button Cell Rev A highlighting the essential subcomponents, (**C**) block diagram of the LMP91000 highlighting its internal features and characteristics (diagram recreated from the chip’s datasheet). Details of the LMP91000 can be found in the chip’s datasheet.

KickStat (Figure 1) includes the LMP91000 (LMP91000SD/NOPB, Texas Instruments, Dallas, TX, USA) potentiostat analog front-end, a SAMD21 (ATSAMD21G18A-MU, Microchip Technology, Chandler, AZ, USA) microcontroller (MCU), 3.3 V linear, low dropout regulator (MIC5504-3.3YM5-TR, Microchip Technology, Chandler, AZ, USA) and a female micro Universal Serial Bus (USB) port (10118192-0001LF, Amphenol FCI, Wallingford, CT, USA) for powering the device and programming the microcontroller. A complete bill-of-materials (BOM) can be found on the online GitHub repository along with full schematics, board layout, and firmware [33]. The schematic (Figure S1) and board layout (Figure S2) are also included in the Supplementary Materials.

The core component of KickStat is the LMP91000 configurable potentiostat featuring an internal voltage reference generator that connects to the counter electrode in a 3-electrode system, an internal transimpedance amplifier (TIA) that converts the current from the working electrode into a voltage, and a series resistance compensation circuit that compensates for the Ohmic drop. The LMP91000 contains an inter-integrated circuit (I2C) serial communication interface allowing the microcontroller to dynamically configure the LMP91000’s settings.

The LMP91000 has built in resistors to control the gain of the TIA in software; however, when measuring currents from the anti-cocaine aptamer, 60 Hz noise, likely coupling in from the USB port and the ambient environment, dominates the electrical signal at these low electrochemical currents. Therefore, we modified the gain of the LMP91000 with an external resistor-capacitor (RC) network in the feedback loop of the TIA (pins 9 and 10 of the LMP91000). This RC network doubled as a first-order, low-pass filter, removing the 60 Hz noise using on-board modifications. Other solutions to remove irradiated noise, such as a Faraday cage, might be impractical for some applications of KickStat such as wearable, continuous monitoring.

The SAMD21 microcontroller includes an internal 10-bit, full-scale digital-to-analog converter (DAC) that, in combination with the LMP91000’s internal voltage reference, is used to dynamically adjust the potential between the working and reference electrodes. The excitation waveforms for each electrochemical test are generated by modulating the voltage from the DAC and internal reference generator of the LMP91000. The waveforms are generated based on the user defined start voltage, end voltage, voltage increment, and scan rate. The firmware then computes the necessary waveforms given the user input parameters. The firmware uses both the DAC and the internal reference generator of the LMP91000 to get as close to the user-desired set voltage as possible. The SAMD21 also includes a native USB interface allowing programming over the USB without using a serial-to-USB bridge, thereby minimizing components. The SAMD21 was loaded with an Arduino bootloader (SAMD21 Mini Breakout Board, SparkFun Electronics, Boulder, CO, USA) and programmed using the Arduino development environment (Arduino IDE v.1.8.8) over the SAMD21’s native USB interface. KickStat draws 11 mA at 3.3 V during full operation, giving the device a power consumption of 36.3 mW. Both the LMP91000 and SAMD21 have shutdown currents down to 2 µA, making KickStat very amenable to wearable and other portable devices that require extremely low power consumption. Such low shutdown currents also make KickStat suitable to incorporate as a subsystem in larger designs.

All components were designed on a 21.6 mm by 20.3 mm rectangular footprint, made of standard FR-4 material, with 1 oz copper, and hot air solder leveling (HASL) finish. All electronic components were sourced from Digi-Key Electronics (Thief River Falls, MN, USA) and have ambient operating temperatures ranging from −40 °C to 85 °C. The circuit boards were fabricated and assembled by PCBWay (Shenzhen, China).

### 2.3. Voltage Bias Generator Mod

Without modifications, the LMP91000 has an internal, 14-point bias circuit allowing for 14 different adjustable potentials between the working and reference electrodes. The voltage reference is set by the LMP91000’s internal bias circuitry and outputs a fraction of the voltage applied to LMP91000’s VREF pin. The available biases are 0%, 1%, 2%, 4%, 6%, 8%, 10%, 12%, 14%, 16%, 18%, 20%, 22%, and 24%. At an operating voltage of 3.3 V, a 14-point setting, provides a voltage resolution of about 66 mV and, at best, 54 mV voltage resolution at the LMP91000’s lowest operating voltage of 2.7 V. Neither of these are sufficient for more advanced techniques and small signals that require a 1 mV voltage resolution.

To improve signal resolution, we augmented the LMP91000’s voltage reference with the SAMD21’s internal 10-bit digital-to-analog converter (DAC) by connecting the output of the DAC to the LMP91000’s VREF pin. The value of the DAC is dynamically adjusted in software based on the desired bias potential for the electrochemical cell. However, changing the voltage at VREF also changes the internal zero of the LMP91000. Subtracting the internal zero from the output of the LMP91000 and then dividing by the value of the feedback impedance results in an accurate measure of the electrochemical current.

The 10-bit DAC has a resolution of 3.3 mV. The LMP91000 has an internal reference generator that can be set to 24% down to 0% of the voltage from the DAC (in increments of 2%). If we multiply the DAC’s 3.3 mV resolution by 24%, we can achieve voltage increments of ~1 mV across the full ±0.792 V excitation range (against an Ag/AgCl reference electrode) with a maximum scan rate of 0.23 V/s.

### 2.4. Noise Measurements

We calculated the input referred noise (measured current with no electrochemical cell) for each of the LMP91000’s built-in gain resistors. The sample rate was set to 60 Hz and sampling time to 60 s. The input referred noise was then determined by measuring the standard deviation of the noise with the limit of detection set to 3 standard deviations. To showcase the device’s high configurability, we also measured the noise when configured with external 2.2 MΩ and 10 MΩ gain resistors. Finally, we measured the noise with a 2.2 MΩ resistor in parallel with a 15 nF capacitor as this configuration was used to measure the signal from the anti-cocaine aptamer (Cocaine Measurements section).

### 2.5. Potassium Ferricyanide Measurements

To validate KickStat for electrochemical measurements, we prepared a 5 mM solution of potassium ferricyanide in PBS (pH 7.4 and room temperature) and compared the device’s response to a high-end commercial potentiostat (VSP-300, Bio-Logic Science Instruments, Seyssinet-Pariset, France) running CV (0 V start and stop potential, in the potential range of −0.2 V to +0.45 V, 1 mV step potential, and at a scan rate of 50 mV/s), CA (−0.2 V to +0.2 V for 40 s with a 10 Hz sampling rate), NPV (−0.2 V to 0.5 V, 10 mV step potential, 50 ms pulse-width, and 200 ms pulse period), and SWV (−0.2 V to +0.5 V, with a 1 mV step potential, 50 mV pulse amplitude, and 31.25 Hz). We used a gold (Au) working electrode (SKU: MF-2014, Bioanalytical Systems, West Lafayette, IN), a silver/silver chloride (Ag/AgCl) reference electrode (SKU: CHI111, CHI Instruments, Austin, TX), and a platinum (Pt) counter electrode (SKU: CHI129, CHI Instruments, Austin, TX). We did not use a Faraday cage in these experiments for either the VSP-300 or KickStat in order evaluate KickStat’s onboard 60 Hz rejection capabilities. We also kept the solutions at room temperature.

### 2.6. Preparing Anti-Cocaine Aptamer-Modified Electrode

The gold working electrode was prepared using a modified version of the protocol presented by Xiao et al. [13]. Briefly, the electrode was cleaned using 1 µm, 0.3 µm and 0.05 µm alumina polish (CH Instruments, Inc. Austin, TX 78738-5012, USA) for approximately 1 min with each polish and rinsed with distilled water in between each polish. The electrode was then sonicated for 5 min in 50:50 (v:v) ethanol:water solution and then rinsed with distilled water. Finally, the electrode was electrochemically cleaned in 0.5 M sulfuric acid using CV scans in the potential range of −0.35 V to 1.4 V at a scan rate of 150 mV/s and 10 mV step size for 7 cycles.

The aptamer binds to the electrode via covalent bonding between the gold electrode and the thiol group on the 5’ end of the aptamer. Before binding can occur, however, the thiol group must first be reduced. The aptamer was prepared by mixing 1 µL of the 250 µM aptamer stock with 2 µL of the 2 mM tris (2-carboxyethyl) phosphine (TCEP) solution. The solution was incubated for 2 h at room temperature in the dark. The aptamer is successfully reduced when the solution turns from blue to colorless. The solution is initially blue due to the MB modification on the 3’ end of the aptamer. However, the blue color fades as TCEP reduces MB to its colorless form, leuco-MB, in addition to reducing the thiol group [13].

The aptamer-TCEP solution was then diluted with 500 µL of PBS to create a 400 nM aptamer solution. The electrode was then incubated with 150 µL of the 400 nM aptamer-TCEP solution for 2 h in the dark at room temperature. Careful timing was necessary to ensure the electrode cleaning step was completed just as the aptamer finished incubating with TCEP to prevent excess wait time between steps. Unnecessary wait times between cleaning and binding steps will increase the exposure of the gold electrode to air, increasing the opportunity for surface oxidation of the electrode which reduces binding of the aptamer to the electrode.

After 2 h of incubation, the electrode was gently rinsed with deionized water for 20 s. Remaining bare gold was blocked by incubating in 150 µL of a 20 µM 6-mercaptohexanol (MCH) solution for 2 h, in the dark at room temperature. After incubation, excess MCH was rinsed off with deionized water.

### 2.7. Cocaine Measurements

The electrode was scanned using CV (7 cycles, −40 mV start and stop potential, from −0.45 V to −20 mV, 1 mV steps, at 50 mV/s) in cocaine-free PBS (pH 7.4 and at room temperature) to assess whether or not the functionalization process was successful. To evaluate the effect of our improved voltage reference generator on the measured signal compared to the LMP91000’s stock generator, we performed the CV scan with steps sizes of 1 mV and 66 mV. Sixty-six (66) mV is the best resolution the LMP91000’s voltage reference generator can achieve given KickStat’s 3.3 V operating voltage without our technique. Then, SWV was used to interrogate the electrode in the presence and absence of cocaine with both KickStat and the VSP-300. The voltage was swept from −0.03 V to −0.5 V with a pulse amplitude of 50 mV, step size of 1 mV, and a frequency of 62.5 Hz in cocaine-free buffer solution and in a 0.5 mM cocaine hydrochloride solution.

Because the measured currents were very low (<1 μA), it was necessary to include an RC filter into the feedback path of the LMP91000’s internal transimpedance amplifier. For CV, a 2.2 MΩ resistor and a 15 nF capacitor were placed in parallel across pins C1 and C2 of the LMP91000 (Figure 1A). For SWV, a 180 kΩ resistor and a 10 nF capacitor were used instead. Each RC network created a first-order, low-pass filter (fc = 4.8 Hz and fc = 88 Hz, respectively), decoupling noise from the measured signal and improving the stability of the amplifier. The cut-off frequency for the RC filter used during SWV was higher than the cutoff frequency used for CV since the SWV signal itself was 62.5 Hz. As with the potassium ferricyanide measurements, we did not use a Faraday cage in this experiment for either the VSP-300 or KickStat in order evaluate KickStat’s on-board 60 Hz rejection capabilities using the RC filter. We also kept the solutions at room temperature.

### 2.8. KickStat Analyst

We equipped KickStat with on-board signal processing capabilities for analyzing our resulting signals, removing the need to export the data for additional analysis. CV and SWV analysis require a peak finding algorithm. Electrochemical peak currents are determined by extrapolating the tangent to the baseline region preceding the redox potential and calculating the peak current relative the tangent line. The tangent to the baseline is determined using the least squared method following Equations (1) and (2) [34]. The tangent line takes the form of a single-variable linear equation where *b_1_* is the slope of the line, *b_0_* is the y-intercept, *x_i_* is the bias potential of the electrochemical cell, and *y_i_* is the measured current at each potential, *x_i_*. The peak current is then determined by finding the maximum difference between the tangent line and the current at potentials around the estimated redox potential (Equation (3)).

Baseline regions and estimated redox potentials for potassium ferricyanide and cocaine sensor were defined based on previous knowledge of the resulting voltammograms. For potassium ferricyanide, the baseline region for the oxidation peak and reduction peaks were −0.185 V to −0.03 V and 0.4 V and 0.3 V respectively. The corresponding peaks were defined in the range of 0.14 V to 0.3 V and 0.17 V to 0.03 V, respectively. The baseline region for the cocaine sensor’s reduction peak was defined in the potential range −0.05 V to −0.2 V. The reduction peak was defined in the potential range −0.315 V to −0.375 V.

(1) $$b_{1}=\frac{\sum_{i=0}^{n}x_{i}y_{i}-\frac{\sum_{i=0}^{n}x_{i}*\sum_{i=0}^{n}y_{i}}{n}}{\sum_{i=1}^{N}{x_{i}}^{2}-\frac{{\left(\sum_{i=0}^{n}x_{i}\right)}^{2}}{n}}$$

(2) $$b_{0}=\frac{\sum_{i=0}^{n}y_{i}-\left(b_{1}*\sum_{i=0}^{n}x_{i}\right)}{n}$$

(3) $$\max\left(y_{i}-\left(b_{0}+b_{1}x_{i}\right)\right)$$

## 3. Results

### 3.1. Noise Measurements

**Figure 2:**
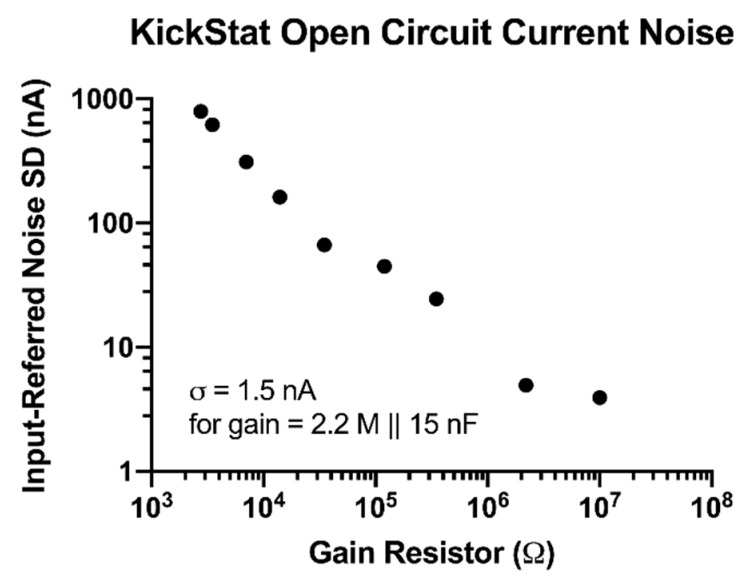
Open circuit current measurements with calculated input-referred noise. Noise decreases as the gain resistor increases.

We observed improved noise performance with increasing gain resistors down to 3.9 nA with a 10 MΩ gain resistor (Figure 2). When we added a 15 nF capacitor in parallel with a 2.2 MΩ gain resistor, we measured a noise level of 1.5 nA giving KickStat an instrumental limit of detection of 4.5 nA (3 times the standard deviation of the noise).

### 3.2. Potassium Ferricyanide

**Figure 3:**
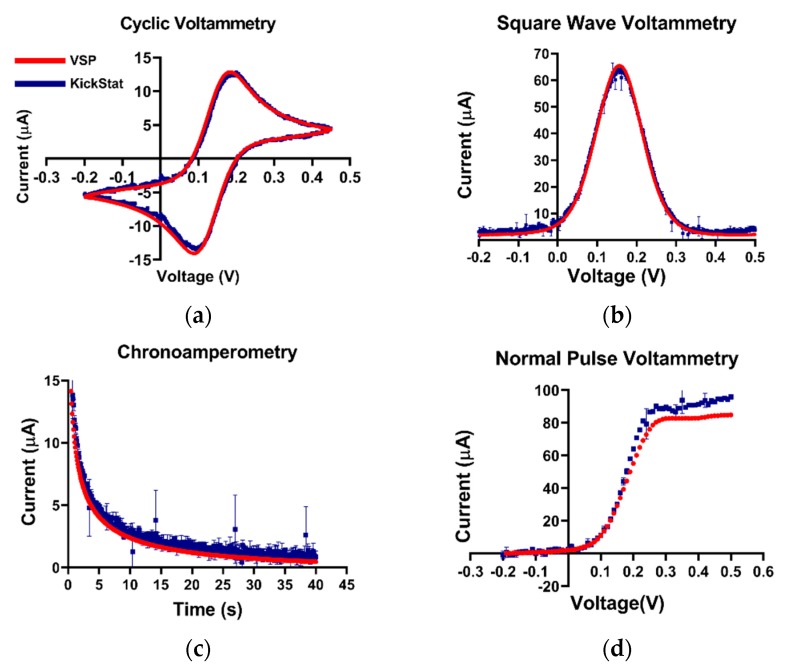
Quantitative comparisons between KickStat (blue) and the commercial device (red) while measuring 5 mM potassium ferricyanide with different electrochemical techniques. (**a**) Cyclic voltammetry, (**b**) square wave voltammetry, (**c**) chronoamperometry, and (**d**) normal pulse voltammetry. Peak values of the current are within 9% for each measurement across each electrochemical technique. Each data point shown for each device is the average of 3 sequential runs. Error bars represent standard deviation and are smaller than the points plotted. Voltages are referenced against an Ag/AgCl reference electrode.

Potassium ferricyanide is a common redox moiety due to its exemplary Nernstian behavior. Therefore, we chose this solution as a benchmark for our device (Figure 3). In the cyclic voltammogram, we observe similar measured anodic and cathodic currents as well as redox potentials from both KickStat and the commercial potentiostat. KickStat reported an anodic current magnitude of 14.39 μA and a cathodic current magnitude of 14.70 μA at 0.2 V and 0.095 V, respectively. The commercial device reported an anodic current magnitude of 15.24 μA and a cathodic current magnitude of 15.28 μA at 0.180 V and 0.089 V, respectively. Overall, KickStat had a root-mean-square deviation (RMSE) of 0.44 μA for CV measurements compared to the commercial potentiostat. In the square wave voltammogram, KickStat reported a current of 58.12 μA, while the commercial device reported 61.99 μA giving KickStat a percent error of 8.1% and an RMSE of 1.23 μA. Both KickStat and the commercial device reported similar responses when running NPV (RMSE of 6.13 μA) and CA (RMSE of 0.63 μA). KickStat slightly overestimated the current in NPV by about 5 μA (6.1%). This is possibly due to the error of the internal TIA gain, which can be as high as 5% according to the datasheet [29]. We noticed that there was slightly more variability in KickStat’s measurements particularly right at the peaks in each voltammogram. Such variability may lead to underestimation or overestimation of the peak current. Further digital smoothing of the data, such as with a Savitzky–Golay filter, could improve our calculations and lower our percent error. Despite this error, we observed better agreement with a commercial potentiostat than others using the LMP91000 have reported [14,22,25] and are able to run more advanced electrochemical techniques, such as SWV and NPV, instead of CV alone.

### 3.3. Electrochemical Response of Cocaine Aptamer

**Figure 4:**
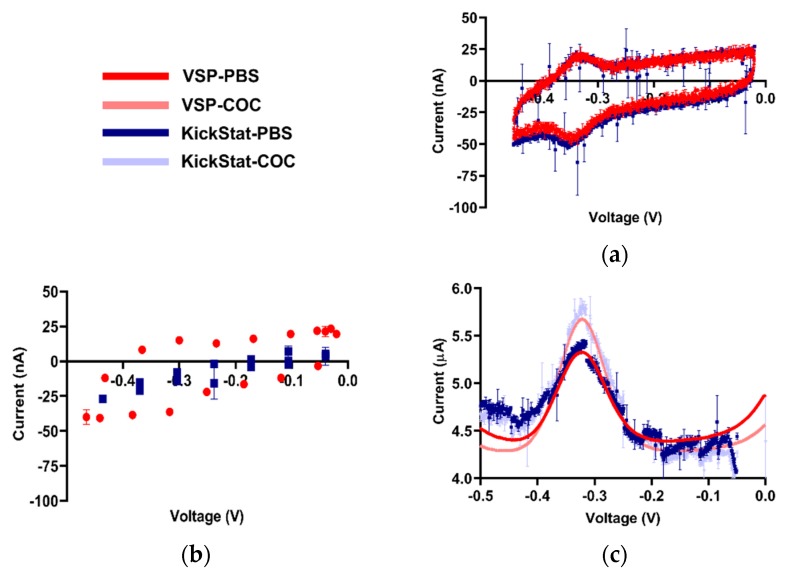
Qualitative comparisons between KickStat (blue) and the commercial device (red) while measuring cocaine biosensor. (**a**) Comparative readout of the cyclic voltammogram for the cocaine aptamer in phosphate-buffered saline (PBS) displaying minimal redox peak separation characteristic of an adsorbed species, indicating successful functionalization of the electrode, (**b**) Cyclic voltammogram with the lower resolution LMP91000 stock voltage reference generator and corresponding points using the commercial device. Peaks are not discernible by eye or by commercial device’s software, making analysis of the electrochemical current virtually impossible, (**c**) Square wave voltammograms in PBS and 0.5 mM cocaine hydrochloride. Data points shown for each device are the average of 3 sequential runs. Error bars represent standard deviation and are smaller than the points plotted in many cases. Voltages are referenced against an Ag/AgCl reference electrode.

After verifying KickStat with potassium ferricyanide, we proceeded with measuring the response of our cocaine biosensor, a more challenging electrochemical species due to low faradaic currents measured from the aptamer (< 1 μA). From the cyclic voltammogram shown in Figure 4a, we observe the standard voltammetric response of an adsorbed species with a reversible electrochemical reaction [35] indicating the electrode was successfully modified. Both KickStat and the commercial device report near zero separation between the oxidation and the reduction peaks (~20 mV) as expected for an adsorbed species [35] and are able to resolve the nA changes in electrochemical currents. However, when utilizing the stock features of the LMP91000 without our improvements (Figure 4b), no peak in the voltammograms was observed. When set to match the voltage resolution of the LMP91000, the commercial potentiostat software was also unable to calculate a peak with so few points. Furthermore, the unmodified LMP91000 current response is very different from that of the commercial device. This poor correlation shows that simply using the stock features of the LMP91000 vastly underestimates the measured current, particularly at electrochemical currents less than 1 μA.

Using both KickStat and the commercial VSP instrument, we measured faradaic currents of 1.04 μA in PBS (Figure 4c) with an RMSE of 0.15 μA. In the presence of 0.5 mM cocaine, this measurement increased to 1.44 μA in KickStat (with an RMSE of 0.17 μA) and 1.43 μA in the commercial instrument. Thus, the response to cocaine was similar for both KickStat and the commercial instrument, each reporting increases in signal of 37.9% (±3.1%) and 37.3% (±0.3%), respectively. Results of a Student’s t-test indicate that the gains measured by both devices are not statistically significantly different from one another (p = 0.75, 2-tailed, unequal variance). These differences in response to 0.5 mM cocaine hydrochloride compared to PBS agree with previous literature reporting 31.4% increase in signal in 0.44 mM cocaine hydrochloride solution when compared to cocaine-free buffer [5].

Of note, KickStat’s response is observably noisier than the commercial device, however, the overall difference in the measured currents with and without cocaine remain the same. Nonetheless, the noise could be further reduced by lowering the cut-off frequency of the RC filter incorporated in the LMP91000’s transimpedance amplifier (as discussed in the methods) and incorporating a sliding average into KickStat’s firmware. We also noticed a difference in baseline between KickStat and the commercial device. Since the faradaic currents that result from the electrochemical process are calculated relative to the baseline, such discrepancies between the two devices did not affect our measured peak currents of interest.

## 4. Discussion

Our results demonstrate that KickStat is comparable to a high-end benchtop potentiostat and is suitable for high-resolution monitoring of aptamer-based biosensors via SWV as well as for general electrochemical measurements: CV, CA, and NPV. KickStat improves upon previous miniaturized potentiostats with its extended electrochemical capabilities and further reduced size. We have skillfully maximized critical features of the LMP91000 by expanding its functionality beyond its stock features while leveraging its integrated design to minimize the overall device footprint. By adding these features, we are able to perform high resolution CV and SWV and to detect aptamer-cocaine binding, a reversible electrochemical reaction. KickStat relies on a single IC, making KickStat more easily incorporated as a subsystem in designs requiring extreme miniaturization and minimal cost. Such advantages are preferred in wearable and other portable applications compared to larger, more expensive module-oriented potentiostats such as the EmStat Pico. Although the EmStat Pico has more features, its larger size (61 × 43 × 16 mm) and higher cost (~1000 Euro/unit) [9] make it more suitable for benchtop units with more relaxed size and cost constraints.

Future work includes adding Bluetooth connectivity as well as enabling electrochemical impedance spectroscopy (EIS), another popular technique for assessing interfacial properties of aptamer-based biosensors. By making the designs, firmware, build instructions, and guidelines for use freely available in an online GitHub repository [33], others can use this miniature platform to extend the capabilities of their own electrochemical biosensor research.

## Supplementary Materials

The following are available online at https://www.mdpi.com/1424-8220/20/8/2407/s1: Figure S1: Full KickStat schematic, Figure S2: KickStat board layout.
