# Supplementary material for: KickStat: A Coin-Sized Potentiostat for High-Resolution Electrochemical Analysis
Source: Sensors (Basel). 2020 Apr 23;20(8):2407. doi: 10.3390/s20082407 (PMC7219484; doi:10.3390/s20082407)
Supplement: Supplementary file 1 [file sensors-20-02407-s001.pdf]

KickStat: A Coin-Sized Potentiostat for High-Resolution Electrochemical Analysis

Orlando S. Hoilett [1], Jenna F. Walker [1], Bethany M. Balash [1], Nicholas J. Jaras [2], Sriram Boppana [1] and Jacqueline C. Linnes [1,*]

[1] Weldon School of Biomedical Engineering, Purdue University, West Lafayette, IN 47907, United States; ohoilett@purdue.edu (O.S.H.); walke327@purdue.edu (J.F.W.); bmdoehrm@purdue.edu (B.M.B.); boppana113@gmail.com (S.B.); jlinnes@purdue.edu (J.C.L.)

[2] School of Electrical and Computer Engineering, Purdue University, West Lafayette, Indiana 47907, United States; njaras24@gmail.com

* Correspondence: jlinnes@purdue.edu; Tel.: +1-765-494-2995

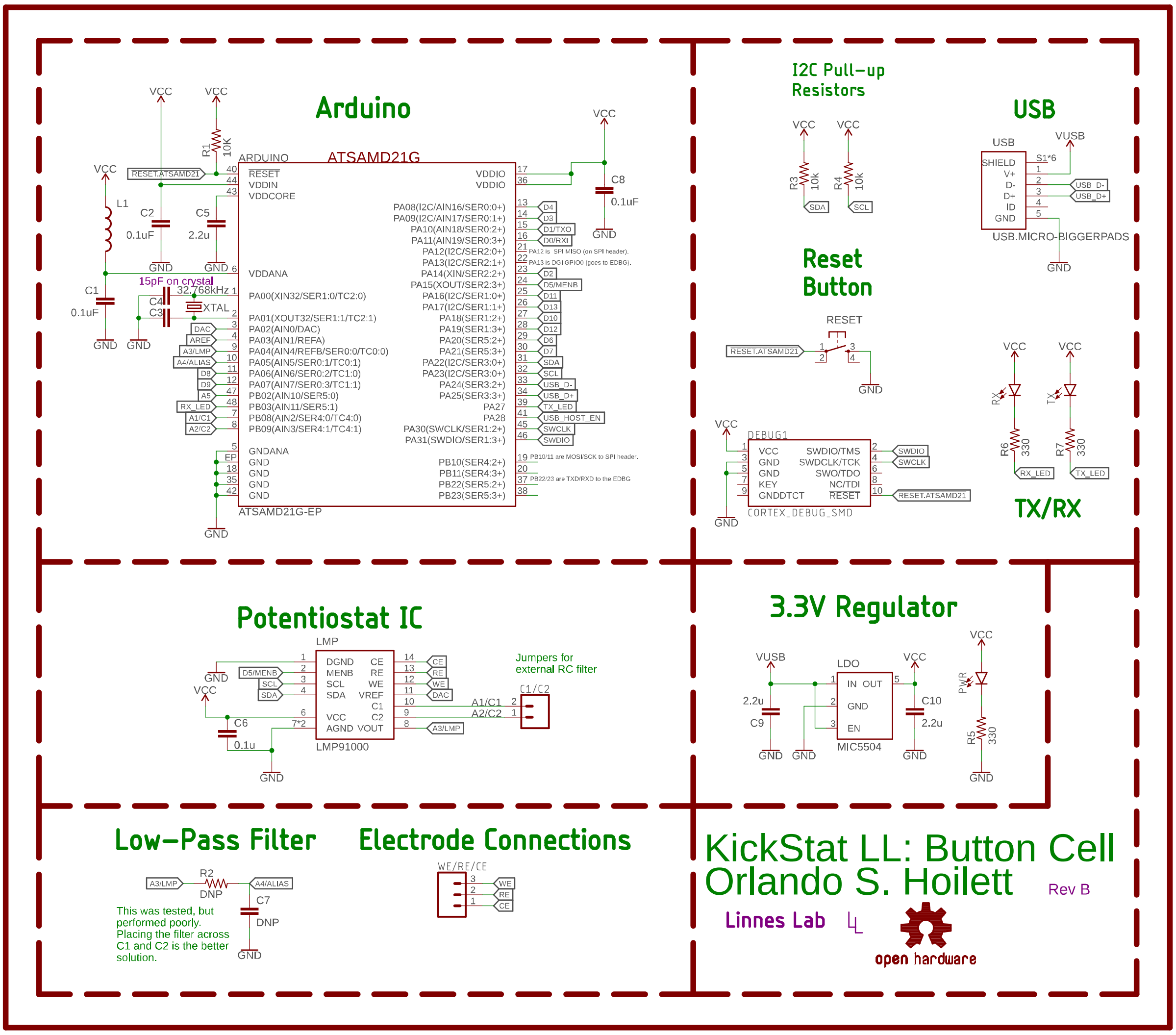

Figure S1. Full KickStat schematic.

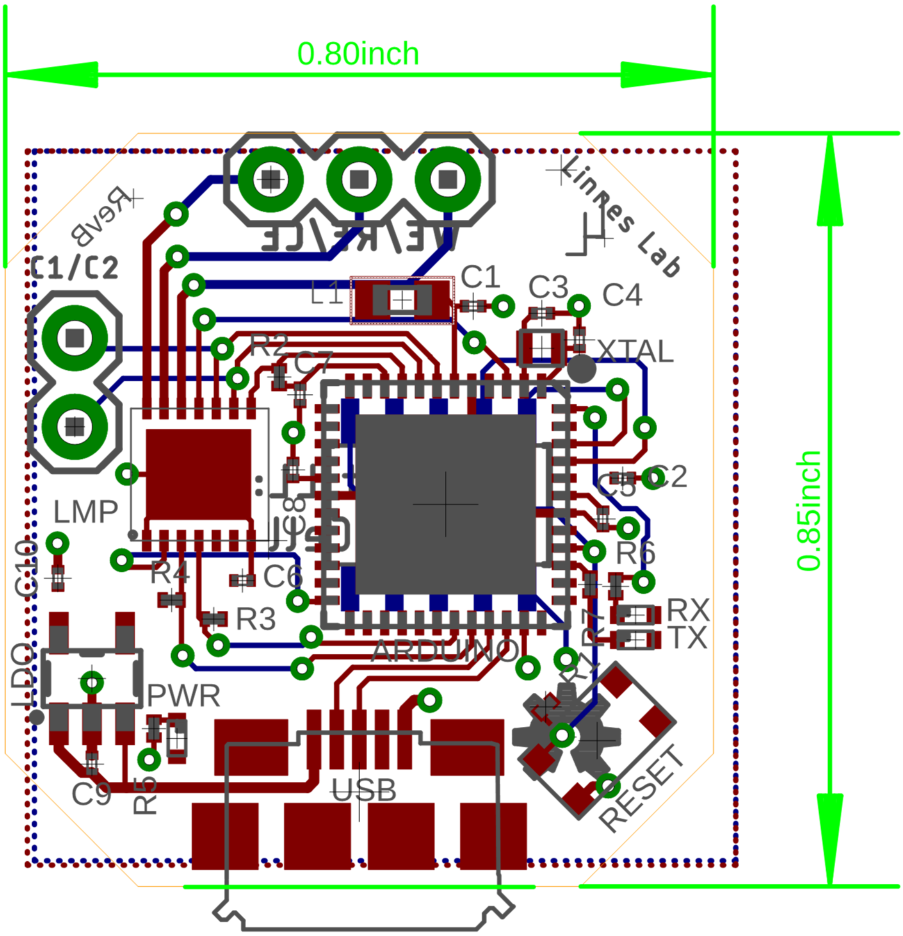

Figure S2. KickStat board layout.
